# Mechanistically guided posterior box isolation using pace-capture resting and threshold-adjusted voltage mapping

**DOI:** 10.1016/j.hroo.2026.02.013

**Published:** 2026-02-23

**Authors:** Koji Kumagai, Yuki Kurose, Kosei Matsuda, Kazushi Kinebuchi, Katsuhiro Ichihashi, Yuhi Hasebe, Kaoru Hasegawa, Takeyoshi Kameyama, Minoru Yambe, Tatsuya Komaru

**Affiliations:** Department of Cardiovascular Medicine, Tohoku Medical and Pharmaceutical University, Miyagi, Japan

**Keywords:** Atrial fibrillation, Posterior wall isolation, Pace-capture testing, Voltage mapping, Catheter ablation

**Key Findings**

▪ A mechanistically guided posterior box isolation strategy integrating pace-capture testing and threshold-adjusted voltage mapping enabled targeted identification of residual posterior wall conduction.
▪ High-output pace-capture testing unmasked functional conduction gaps not evident on conventional voltage mapping alone.
▪ Threshold-adjusted voltage mapping localized discrete high-voltage myocardial islands within the posterior wall.
▪ This hybrid strategy achieved posterior box isolation with a limited number of additional lesions.
▪ Short-term arrhythmia-free survival was higher with the mechanistically guided approach than a conventional activation-guided strategy.

## Introduction

Durable posterior wall isolation remains challenging in atrial fibrillation (AF) ablation despite its strong mechanistic rationale.^1^ Large randomized trials have failed to demonstrate incremental benefit when empirical posterior wall ablation is added to pulmonary vein isolation (PVI), suggesting that indiscriminate posterior wall homogenization may not adequately address critical residual conduction pathways.^2^^,^^3^ We evaluated an exploratory, mechanistically guided strategy integrating functional lesion assessment and substrate-based mapping to achieve posterior box isolation (POBI) after PVI.

This single-center retrospective study included 55 consecutive patients undergoing catheter ablation for AF. After PVI, patients underwent either a hybrid pace capture– and voltage-guided POBI strategy (hybrid group, n = 31) or a conventional activation map–guided approach (conventional group, n = 24). The hybrid strategy was introduced in October 2021 and applied to consecutive patients thereafter.

**Figure 1:**
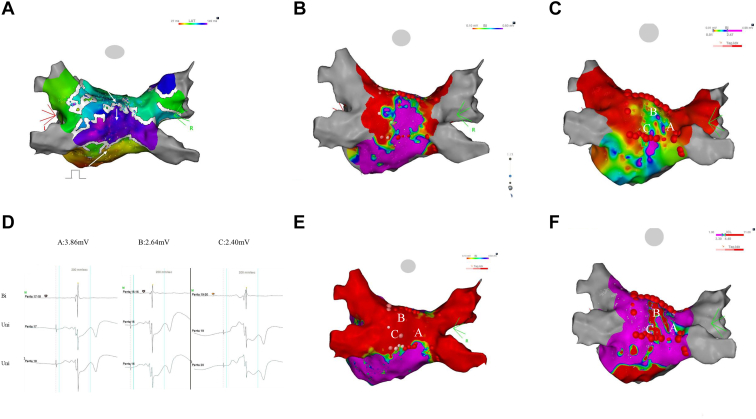
Mechanistically guided posterior box isolation using pace-capture testing and threshold-adjusted voltage mapping. **(A)** Local activation time mapping during coronary sinus pacing demonstrates propagation across the roof line into the posterior wall despite apparently continuous linear lesions. **(B)** Conventional bipolar voltage mapping shows a broad preserved voltage area (≥0.5 mV) within the posterior box. **(C)** Threshold-adjusted bipolar voltage mapping reveals discrete high-voltage islands. **(D)** Representative bipolar and unipolar electrograms recorded at high-voltage islands. **(E)** Targeted focal ablation at high-voltage islands completes posterior box isolation, confirmed by the absence of local electrograms (<0.1 mV) and noncapture during high-output pacing (10 V/1 ms). **(F)** High-voltage islands overlap with fractionated signal areas identified by interval confidence level analysis. Bi = bipolar; Uni = unipolar.

After PVI, roof and floor lines were created to encircle the posterior wall. The functional integrity of these linear lesions was assessed using high-output pacing (10 V/1 ms).^4^ Sites with persistent capture underwent targeted reinforcement adjacent to the line to avoid thermal stacking. Posterior wall bipolar voltage mapping was then performed with dynamically adjusted thresholds to identify discrete high-voltage islands (≥0.5 mV).^5^ When present, focal ablation was applied. POBI was confirmed using a composite endpoint defined as the absence of capture during high-output pacing, a bipolar voltage of <0.1 mV within the posterior box, and the absence of early activation on activation mapping, rather than formal entrance/exit block testing (Figure 1).

The research was approved by the suitably constituted ethics committee of Tohoku Medical and Pharmaceutical University, and it conformed to the provisions of the Declaration of Helsinki. All patients provided a written informed consent. Patients were followed with scheduled clinic visits, surface electrocardiograms, and 24-hour Holter monitoring. Atrial arrhythmia recurrence was defined as documented AF, atrial tachycardia, or atrial flutter lasting ≥30 seconds beyond a 3-month blanking period.

The hybrid and conventional groups were comparable with respect to age, sex, distribution, and AF type. Complete POBI was achieved in all hybrid-group patients and in 91.7% of the conventional group. Within the hybrid group, roof and floor lines alone achieved isolation in 12 patients (38.7%), whereas 19 patients (61.3%) required additional focal ablation guided by pace-capture testing and threshold-adjusted voltage mapping. The number of additional lesions was limited (2.9 ± 3.2). Preserved high-voltage islands were predominantly located in the central posterior wall and frequently colocalized with fractionated signal areas (Figure 1).

During a mean follow-up of 8.4 months, arrhythmia-free survival was higher in the hybrid group than in the conventional group (87.1% vs 50.0%; log-rank *P* = .022). Within the hybrid group, atrial arrhythmia recurrence occurred in 1 patient treated with roof and floor lines alone and in 3 patients who required additional focal ablation. In sensitivity analyses accounting for adjunctive ablation procedures and propensity score–based adjustment, the direction of the association between the hybrid strategy and arrhythmia-free survival remained unchanged.

This exploratory analysis suggests that combining functional lesion assessment with threshold-adjusted substrate mapping may overcome key limitations of empirical posterior wall isolation. High-output pace-capture testing can unmask residual conduction not evident on voltage attenuation alone,^4^ whereas threshold-adjusted voltage mapping enables localization of preserved myocardial bundles.^5^

The limitations include the retrospective, single-center design, small sample size, short follow-up, limited rhythm monitoring, and lack of remapping. Residual confounding and temporal bias cannot be excluded; therefore, these findings should be regarded as hypothesis generating.

## Conclusion

A mechanistically guided POBI using pace-capture testing and threshold-adjusted voltage mapping enabled targeted posterior wall modification with fewer lesions and improved short-term arrhythmia outcomes vs a conventional approach.

## Disclosures

The authors have no conflicts of interest to disclose.

## References

[1] Calkins H., Hindricks G., Cappato R. (2017). 2017 HRS/EHRA/ECAS/APHRS/SOLAECE expert consensus statement on catheter and surgical ablation of atrial fibrillation. Heart Rhythm.

[2] Kistler P.M., Chieng D., Sugumar H. (2023). Effect of catheter ablation using pulmonary vein isolation with vs without posterior left atrial wall isolation on atrial arrhythmia recurrence in patients with persistent atrial fibrillation: the CAPLA randomized clinical trial. JAMA.

[3] Tonko J.B., Silberbauer J., Mann I. (2023). How to ablate the septo-pulmonary bundle: a case-based review of percutaneous ablation strategies to achieve roof line block. Europace.

[4] Andrade J.G., Pollak S.J., Monir G. (2013). Pulmonary vein isolation using a pace-capture-guided versus an adenosine-guided approach: effect on dormant conduction and long-term freedom from recurrent atrial fibrillation--a prospective study. Circ Arrhythm Electrophysiol.

[5] Rolf S., Kircher S., Arya A. (2014). Tailored atrial substrate modification based on low-voltage areas in catheter ablation of atrial fibrillation. Circ Arrhythm Electrophysiol.

